# Efficacy of olanzapine long-acting injection in patients with acutely exacerbated schizophrenia: an insight from effect size comparison with historical oral data

**DOI:** 10.1186/1471-244X-12-51

**Published:** 2012-05-30

**Authors:** Holland C Detke, Fangyi Zhao, Michael M Witte

**Affiliations:** 1 Eli Lilly and Company, Indianapolis, IN, USA; 2 Lilly USA, LLC, Indianapolis, IN, USA

**Keywords:** Olanzapine, Long-acting injection, Pamoate, Haloperidol, Effect size, Onset, Pharmacokinetics

## Abstract

**Background:**

To treat acute schizophrenia, a long-acting injectable antipsychotic needs a rapid onset of action and therapeutic profile similar to that of oral agents. The present post-hoc analyses compared results from a randomized, double-blind, placebo-controlled trial of olanzapine long-acting injection (LAI) for acute schizophrenia with those observed in similarly designed trials of oral olanzapine.

**Methods:**

Six-week results from the olanzapine LAI study (N = 404) were compared with those of 3 oral studies (study 1: olanzapine vs. haloperidol vs. placebo [N = 335]; study 2: olanzapine vs. haloperidol vs. low-dose olanzapine [N = 431]; study 3: olanzapine vs. placebo vs. low-dose olanzapine [N = 152]). All patients had baseline Brief Psychiatric Rating Scale (BPRS) scores ≥24 (0–6 scale). Six-week effect sizes were calculated. Efficacy onset, pharmacokinetics, discontinuations, weight gain, and extrapyramidal symptoms were also assessed.

**Results:**

At 6 weeks, mean BPRS scores decreased by 14 to 15 points for olanzapine LAI (405 mg/4 weeks, 210 or 300 mg/2 weeks), by 8 to 16 for oral olanzapine (10 ± 2.5 or 15 ± 2.5 mg/day), and by 12 to 13 for haloperidol (15 ± 5 mg/day). For those same dose groups, effect sizes vs. placebo for the BPRS were 0.7 to 0.8 for olanzapine LAI, 0.5 to 0.7 for oral olanzapine, and 0.6 for haloperidol. The first statistically significant separation from placebo on the BPRS occurred at 3 days for the olanzapine LAI groups and at 1 week for oral olanzapine and haloperidol (15 ± 5 mg/day) in oral study 1 although as late as week 6 for the 10-mg/day olanzapine dose in oral study 3. Olanzapine concentrations were similar across studies. Weight gain ≥7% of baseline occurred in up to 35% of olanzapine LAI and oral patients versus up to 12% of haloperidol and placebo patients. Extrapyramidal symptoms were lowest in the olanzapine LAI groups and significantly greater in the haloperidol groups. No post-injection delirium/sedation syndrome events occurred in the olanzapine LAI study.

**Conclusions:**

Patients treated acutely with olanzapine LAI showed a similar pattern of improvement to that seen historically with oral olanzapine. With the exception of injection-related adverse events, the efficacy and tolerability profile of olanzapine LAI is similar to oral olanzapine.

**Trial registration:**

ClinicalTrials.gov ID; URL: http://http//www.clinicaltrials.gov/: NCT00088478; ClinicalStudyResults.org ID; URL: http://www.clinicalstudyresults.org/: 917, 978, 982, and 5984.

## Background

In the treatment of patients with acute symptoms of schizophrenia, second generation oral antipsychotics have been the treatment of choice, and one of the standards of care is oral olanzapine [1]. In the United States, the use of long-acting formulations have been reserved for patients who have difficulty complying with oral regimens during maintenance treatment [2-4], and little information is available about their use for patients with acute symptoms of schizophrenia [5]. Given the high frequency of insufficient medication adherence among patients with schizophrenia [6-8], it could be clinically advantageous to be able to initiate a long-acting depot antipsychotic during an acute exacerbation. To benefit patients with acute symptoms, a long-acting antipsychotic formulation needs a rapid onset of action, combined with a therapeutic profile similar to that of second generation oral agents.

Olanzapine long-acting injection (LAI) has demonstrated efficacy in acutely ill patients [9] and has demonstrated similarity to oral olanzapine in terms of maintenance of effect and safety during longer-term treatment [10]; several recent reviews are available [11-13]. However, no direct comparisons of oral and LAI olanzapine have been done within the acute phase of treatment. In order to address this question, a post-hoc analysis of effect sizes was conducted to compare efficacy results from the acute trial of olanzapine LAI [9] with historical data from 3 similarly designed trials of oral olanzapine for the acute treatment of schizophrenia [14-16]. The 3 oral studies were randomized, controlled trials using either placebo [14,16] or a non-therapeutic dose of oral olanzapine (1 mg/day) as the reference group [15,16]. In addition, oral haloperidol was used as an active comparator in 2 of these oral olanzapine trials [14,15], thus potentially allowing for further clinical context for the olanzapine LAI findings. As in the acute olanzapine LAI trial, the oral trials required patients to be acutely symptomatic, and mean Brief Psychiatric Rating Scale (BPRS) scores (0–6 scale) were similar across all 4 studies, in the range of 37 to 43, indicating markedly ill patient populations. While not providing the degree of control possible in a direct head-to-head comparison, the use of effect sizes allows for useful comparisons of efficacy data across trials that employ similar designs and patient populations. In addition to evaluating effect sizes, we examined onset of efficacy, defined as the time of first statistical separation from placebo or a non-therapeutic dose of olanzapine. Finally, we also provide indirect comparison of key tolerability measures across these trials.

## Methods

### Study criteria

To be included in the analyses, studies had to be randomized, double-blind, fixed- or semi-fixed-dose, placebo-controlled and/or employing a low non-therapeutic reference dose as control group, at least 6 weeks in length, and conducted in acutely ill patients with schizophrenia. All were also previously submitted to the United States Food and Drug Administration as part of the submission for approval for the treatment of schizophrenia. The 4 studies meeting these criteria were olanzapine LAI study F1D-MC-HGJZ [9], oral study F1D-MC-HGAD [14], oral study F1D-EW-E003 [15], and oral study F1D-MC-HGAP [16] (See Table 1). All patients were diagnosed with schizophrenia using then-current *Diagnostic and Statistical Manual of Mental Disorders* (DSM) criteria and had to meet minimum BPRS criteria (at least ≥24) to ensure that patients were acutely ill at study entry. Three of the studies compared the respective formulation of olanzapine (LAI or oral) with placebo [9,14,16]; two of the studies employed a non-therapeutic reference dose of oral olanzapine (1 mg/day) [15,16]. In all 4 studies, patients were required to be inpatients for at least the first 2 weeks of the study and could potentially become outpatients thereafter if clinically appropriate. The olanzapine LAI study excluded patients who were known to be treatment-resistant to olanzapine but did not exclude other treatment-resistant patients. Oral studies 1 and 2 excluded patients who were known to be clear non-responders to neuroleptic treatment. Oral study 3 excluded patients who had failed to show minimal clinical response to sufficient doses of at least 3 different neuroleptics from 3 different chemical classes or to a dose of 400 mg/d of clozapine for at least 6 weeks.

**Table 1:** A summary of the olanzapine long-acting injection and oral olanzapine studies analyzed

**Study**	**Dates Conducted**	**Primary Objective**	**Design**	**Treatments, Doses, and Regimen**	**N**	**Diagnosis and Inclusion Criteria**
**OLZ LAI**[9]	Jun 2004 –Apr 2005	Efficacy vs. placebo (PANSS Total)	2 – 7 day washout	**OLZ LAI**	404	DSM-IV schizophrenia
			8 weeks	405 mg/4 weeks		BPRS score ≥30 (0 – 6 scale)
			Randomized,	210 mg/2 weeks		
			double-blind	300 mg/2 weeks		
				**IM placebo/2 weeks**		
**Oral OLZ, Study 1**[14]	Oct 1991 – Nov 1996	Efficacy vs. placebo and HAL (BPRS)	4 – 7 day placebo lead-in	**OLZ**	335	DSM-III-R schizophrenia with acute exacerbation
				5 ± 2.5 mg/day		
			6 weeks	10 ± 2.5 mg/day		BPRS score ≥24 (0 – 6 scale)
			Randomized,	15 ± 2.5 mg/day		
			double-blind	**HAL:** 15 ± 5 mg/day		
				**Placebo**		
**Oral OLZ, Study 2**[15]	Nov 1991 – Feb 1997	Efficacy vs. HAL (BPRS)	4 – 7 day placebo lead-in	**OLZ**	431	DSM-III-R schizophrenia with acute exacerbation
				5 ± 2.5 mg/day		
			6 weeks	10 ± 2.5 mg/day		BPRS score ≥24 (0 – 6 scale)
			Randomized,	15 ± 2.5 mg/day		CGI-S score ≥4
			double-blind	1 mg/day		
				**HAL:** 15 ± 5 mg/day		
**Oral OLZ, Study 3**[16]	Jul 1993 – Jan 1997	Efficacy vs. Placebo (BPRS)	4 – 9 day placebo lead-in	**OLZ**	152	DSM-III-R schizophrenia with acute exacerbation
				1 mg/day		
			6 weeks	10 mg/day		BPRS score ≥24 (0 – 6 scale)
			Randomized, double-blind	**Placebo**		

Abbreviations: BPRS: Brief Psychiatric Rating Scale; CGI-S: Clinical Global Impressions – Severity; DSM: Diagnostic and Statistical Manual (versions III-R: revised; and IV); OLZ: olanzapine; HAL: haloperidol; OLZ LAI: olanzapine long-acting injection; PANSS: Positive and Negative Syndrome Scale

The BPRS total scores and/or the Positive and Negative Syndrome Scale (PANSS) total scores were used to assess efficacy. Pharmacokinetic sampling was conducted in all patients at the end of the visit, just prior to the next dose for oral patients or just prior to the next injection for LAI patients when at an injection visit. LAI patients also had assessments taken periodically between injections. Heparinized plasma samples were analyzed for olanzapine using a validated high-performance liquid chromatography method at the same centralized laboratory across all 4 studies. For the LAI study, samples collected >1 day before or after the intended visit interval were excluded from analysis. Safety measures included mean change in weight from baseline to endpoint and the incidence of treatment-emergent weight gain ≥7% from baseline. Treatment-emergent Parkinsonism was defined as a Simpson-Angus [17] total baseline score ≤3 and a post-baseline score >3 at anytime. Treatment-emergent akathisia was defined as a Barnes Akathisia Rating Scale [18] baseline global score <2 and a post-baseline score ≥2 at anytime. Patients were allowed to take benzodiazapines for insomnia, anxiety, or agitation (≤2 mg/day lorazepam equivalents in the olanzapine LAI study, and ≤10 mg/day in the 3 oral studies). Anticholinergic use for extrapyramidal symptoms was permitted (≤6 mg/day biperiden equivalents in all 4 studies), but prophylatic use was prohibited. No oral antipsychotic supplementation was allowed in the olanzapine LAI study. All 4 studies were conducted in accordance with the ethical principles of the Declaration of Helsinki. Protocols were conducted consistent with good clinical practices and all applicable laws and regulations in each region. Ethical Review Boards approved each protocol before investigators initiated the trials. All patients or their legal representative signed an informed consent before participation in the trial. Detailed information about each study has been published elsewhere [9,14-16].

### Statistical analyses

The data in each of the 4 studies were analyzed on an intent-to-treat basis, and analysis results from each study are presented separately here. Standard baseline characteristics were summarized for all randomized patients. Mean changes from baseline to endpoint were analyzed using the last-observation-carried-forward approach. Differences between groups in continuous data within each study were assessed using analysis of variance, with treatment, investigator (or country), and/or treatment-by-investigator (or treatment-by-country) interaction as fixed factors. Differences between groups in categorical data were assessed with Fisher’s exact test or Pearson’s chi-square test. All comparisons were conducted at a two-sided alpha level of 0.05 without adjustment for multiplicity. Onset of efficacy was defined as the first time point at which a treatment group achieved statistical superiority to the reference group (placebo or low non-therapeutic dose) followed by statistical superiority at all subsequent visits.

Effect size for each treatment group was calculated against placebo or the non-therapeutic dose of oral olanzapine (1 mg/day) as the reference group. Effect size was calculated as the difference of mean changes in efficacy measures (BPRS and PANSS total scores) of the treatment group and the reference group (placebo or oral olanzapine 1 mg/day), divided by their pooled standard deviations. Because the oral olanzapine studies were only 6 weeks in length while the olanzapine LAI study was 8 weeks in length, only the first 6 weeks of data from the olanzapine LAI study were used for the between-study efficacy comparison; however, pharmacokinetic and safety comparisons were not adjusted for time in treatment.

## Results

### Baseline symptom severity

Patient demographics and baseline symptom severity were similar across the 4 studies (Table 2). Patients were mostly male (64–88%) and Caucasian (56–86%), with mean ages of 36 to 41 years.

**Table 2:** Patient demographics and baseline illness characteristics

**Characteristic**	**OLZ LAI Study** (N = 404)	**Oral Study 1** (N = 335)	**Oral Study 2** (N = 431)	**Oral Study 3** (N = 152)
**Male, n (%)**	285 (70.5)	294 (87.8)	275 (63.8)	110 (72.4)
**Caucasian, n (%)**	226 (55.9)	230 (68.7)	372 (86.3)	104 (68.4)
**Mean Age, years (SD)**	40.8 (11.2)	36.0 (9.4)	35.5 (10.7)	37.6 (9.2)
**Mean Age of Disease Onset, years (SD)**	23.4 (8.2)	22.0 (5.8)	24.1 (7.7)	21.7 (5.7)
**Median Length of Current Episode, days**	39	30	32	40
**Mean PANSS Total Score (SD)**	101.0 (15.6)	--	103.3 (18.4)	98.2 (17.7)
**Mean BPRS Total (SD)**	40.9 (8.9)	41.5 (11.0)	40.7 (10.6)	38.0 (9.0)

Abbreviations: *BPRS*: Brief Psychiatric Rating Scale; *OLZ LAI*: olanzapine long-acting injection; *PANSS*: Positive and Negative Syndrome Scale; *SD*: standard deviation

### Efficacy assessments

**Figure 1:**
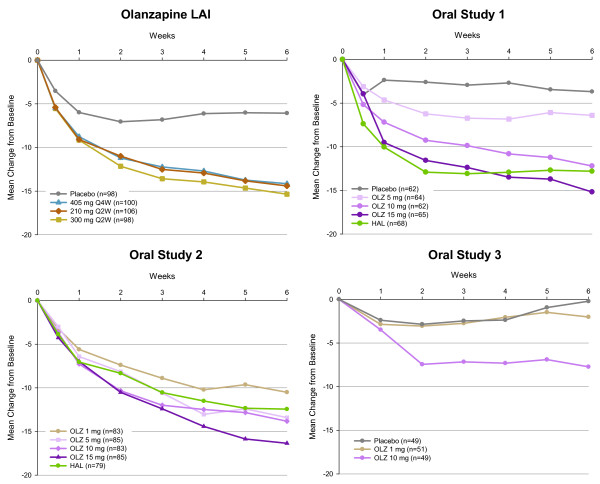
**Mean changes in BPRS scores during six weeks of treatment in olanzapine long-acting injection and oral olanzapine studies of acute schizophrenia**. In the LAI study, all 3 olanzapine LAI treatment groups showed statistically greater reductions vs. placebo by day 3 (*p* = .05) and for the remainder of the study. In oral olanzapine study 1, the 10- and 15-mg/day olanzapine groups and the haloperidol group showed statistically greater reductions vs. placebo at week 1 and beyond (*p* = .05). In oral study 2, no groups separated statistically from the 1-mg reference dose although the overall pattern of symptom reductions appeared similar to those in the other studies. In oral olanzapine study 3, the 10-mg/day treatment group showed a significant difference vs. placebo at week 6 (*p* = .04).

Weekly reduction in symptoms assessed by the BPRS in each of the 4 studies over 6 weeks are shown in Figure 1. Three of the 4 studies also evaluated symptom changes with the PANSS (olanzapine LAI study, oral study 2, and oral study 3), and those results are shown in Additional file 1: Figure S1.

#### Effect size

Table 3 shows the effect sizes for the BPRS and PANSS total assessments for each active treatment group in all 4 studies. Most importantly, the placebo-controlled effect sizes for treatment with olanzapine LAI were comparable to the effect sizes for treatment with oral olanzapine seen in each of the 3 studies. Effect sizes in oral olanzapine study 2 were lower, but this is due to the fact that the 1-mg/day oral olanzapine treatment group was used as the reference group instead of placebo.

**Table 3:** Effect sizes for olanzapine oral or long-acting injection vs. placebo or non-therapeutic olanzapine (1 mg)

**Study and Treatment**	**N**	**Mean Modal Dose/ Corresponding Daily Dose (mg/day)**	**PANSS Total LS Mean Change**	**PANSS Total Effect Size**^**a**^**(category)**^**b**^	**BPRS Total LS Mean Change**	**BPRS Total Effect Size**^**a**^**(category)**^**b**^
**OLZ LAI Study**						
405 mg/4 weeks olanzapine LAI	100	14.5	−22.5	.75 (moderate)	−14.2	.71 (moderate)
210 mg/2 weeks olanzapine LAI	106	15.0	−22.8	.80 (large)	−14.4	.77 (moderate)
300 mg/2 weeks olanzapine LAI	100	21.4	−24.8	.88 (large)	−15.4	.82 (large)
vs. Placebo	98	0.0	−8.7	--	−6.1	--
**Oral Study 1**						
5 ± 2.5 mg/day olanzapine	65	6.6	--	--	−6.4	.17 (small)
10 ± 2.5 mg/day olanzapine	64	11.6	--	--	−12.2	.50 (moderate)
15 ± 2.5 mg/day olanzapine	69	16.3	--	--	−15.2	.67 (moderate)
15 ± 5.0 mg/day haloperidol	69	16.4	--	--	−12.8	.57 (moderate)
vs. Placebo	68	0.0	--	--	−3.7	--
**Oral Study 2**						
5 ± 2.5 mg/day olanzapine	87	6.7	−21.4	.17 (small)	−13.4	.19 (small)
10 ± 2.5 mg/day olanzapine	86	11.3	−22.7	.20 (small)	−13.8	.19 (small)
15 ± 2.5 mg/day olanzapine	89	16.4	−26.7	.37 (moderate)	−16.4	.38 (moderate)
15 ± 5.0 mg/day haloperidol	81	17.6	−20.0	.12 (small)	−12.4	.12 (small)
vs. 1.0 mg/day olanzapine	88	0.0	−16.8	--	−10.5	--
**Oral Study 3**						
10 mg/day olanzapine (vs. placebo)	50	10.0	−12.3	.71 (moderate)	−7.7	.60 (moderate)
10 mg/day olanzapine (vs. 1 mg)	50	10.0	−12.3	.48 (moderate)	−7.7	.45 (moderate)
1 mg/day olanzapine (vs. placebo)	52	1.0	−1.9	.22 (moderate)	−2.0	.14 (small)
Placebo	50	0.0	2.8	--	−0.2	--

^a^ All effect sizes were calculated from baseline-to-endpoint LOCF mean change scores at 6 weeks of treatment (OLZ LAI study was 8 weeks, whereas the 3 oral studies were only 6 weeks)

^b^ Effect sizes were categorized using Cohen’s general effect size thresholds [19]

Abbreviations: BPRS: Brief Psychiatric Rating Scale; LOCF: last observation carried forward; LS: least squares; OLZ LAI: olanzapine long-acting injection; PANSS: Positive and Negative Syndrome Scale

#### Onset of efficacy

Onset of efficacy was demonstrated by all 3 doses of olanzapine LAI vs. placebo starting at day 3 (*p* < .05) on the BPRS. On the PANSS, improvement vs. placebo began at day 3 for two of the olanzapine LAI dose groups (405 mg/4 weeks and 300 mg/2 weeks; *p* < .05) and at week 1 for the other dose group (210 mg/2 weeks; *p* < .01). In oral study 1, the medium and high olanzapine dose groups (10 ± 2.5 and 15 ± 2.5 mg/day) and the haloperidol group (15 ± 5 mg/day) all demonstrated onset of efficacy compared with placebo starting at week 1 (*p* < .05) on the BPRS; the low dose olanzapine group (5 ± 2.5 mg/day) separated from placebo only at week 2 of treatment (*p* < .05). In oral study 2, although a similar pattern of symptom reduction was seen in the active groups, no groups separated statistically from the very low reference dose comparator (1 mg/day olanzapine) at any time. In oral study 3, onset of efficacy for the 10-mg/day olanzapine group began at the 6-week endpoint vs. placebo on the BPRS (*p* < .05) but began starting at week 2 on the PANSS (*p* < .05).

### Olanzapine plasma concentrations

The expected therapeutic range for olanzapine plasma concentrations for within-label doses of olanzapine (oral or LAI) has been reported as approximately 5 to 73 ng/mL, representing the 10^th^ percentile for the lowest within-label dose and the 90^th^ percentile for the highest within label dose [20]. In the LAI study, mean concentrations (ng/mL) were in the therapeutic range as early as day 3, with means of 9.1 (SD = 8.6), 17.2 (SD = 14.7), and 15.6 (SD = 13.3) for the 210-mg/2 week, 405-mg/4 week, and 300-mg/2 week groups, respectively. Table 4 presents olanzapine plasma concentrations across all studies at study endpoint. Although study endpoint for the olanzapine LAI study was at 8 weeks (vs. 6 weeks for the oral studies), endpoint results represent the best comparison as these indicate trough levels for each of the formulations and dosage regimens, including the 4-week LAI dosage which would otherwise not be at trough level at 6 weeks.

**Table 4:** Olanzapine plasma concentrations at study endpoint for the olanzapine long-acting injection and olanzapine oral studies

**Study and Treatment**	**n**	**Mean at endpoint (ng/mL)**	**SD**	**Minimum**	**Maximum**
**OLZ LAI Study**					
405 mg/4 weeks olanzapine LAI	39	13.4	7.8	5.9	56.0
210 mg/2 weeks olanzapine LAI	55	18.3	9.4	5.5	44.7
300 mg/2 weeks olanzapine LAI	56	24.3	11.6	9.3	87.6
**Oral Study 1**					
5 ± 2.5 mg/day olanzapine	63	10.3	8.0	0.4	36.9
10 ± 2.5 mg/day olanzapine	62	18.4	12.1	0.4	63.2
15 ± 2.5 mg/day olanzapine	63	30.6	22.7	0.5	108.1
**Oral Study 2**					
1 mg/day olanzapine	75	1.7	1.9	0.3	16.0
5 ± 2.5 mg/day olanzapine	78	9.1	5.9	0.5	37.0
10 ± 2.5 mg/day olanzapine	76	16.8	11.5	0.3	64.3
15 ± 2.5 mg/day olanzapine	75	26.9	19.2	1.2	99.8
**Oral Study 3**					
1 mg/day olanzapine	51	1.5	0.8	0.0	3.2
10 mg/day olanzapine	48	20.7	17.1	0.3	93.0

Abbreviations: *n*: number of patients with evaluable samples; *OLZ LAI*: olanzapine long-acting injection; *SD*: standard deviation

### Weight gain

The safety results from the olanzapine LAI study show similar patterns of weight gain for patients in the 3 olanzapine LAI treatment groups as that reported for the therapeutic treatment groups in oral olanzapine studies 1, 2, and 3. At study endpoint, mean weight increased by 2.8–3.9 kg for olanzapine LAI (*p* ≤ .001 vs. placebo for all LAI doses), 1.7–3.6 kg for oral olanzapine (study 1: *p* < .05 vs. placebo for all oral olanzapine doses and *p* < .05 vs. haloperidol for the medium and high olanzapine doses; study 2: *p* < .001 vs. 1 mg oral and *p* < .01 vs. haloperidol for the medium and high olanzapine doses; study 3: *p* < .05 vs. 1 mg and placebo), and −0.4–0.9 kg for haloperidol (not significant vs. placebo or 1 mg/day olanzapine). Weight gain ≥7% of baseline was statistically significantly greater in the olanzapine groups, but comparable between the studies for olanzapine-treated patients: olanzapine LAI vs. placebo (24%–35% vs. 12%, *p’s* < .05); study 1 oral olanzapine (28%–34%) vs. placebo (3%, p’s < .001) and haloperidol (12%, *p* < .05); study 2 oral olanzapine (19%–35%) vs. 1 mg oral (13%, *p’s* < .05) and haloperidol (4%, *p* < .05); and study 3 oral olanzapine (20%) vs. 1 mg oral (6%, *p* < .05) and placebo (2%, *p* < .01).

### Extrapyramidal symptoms

Overall incidence of treatment-emergent extrapyramidal symptoms was lower across all groups in the LAI study than in the older oral studies, including patients in the placebo and non-therapeutic control groups, but was markedly lower for the LAI and oral olanzapine groups than the haloperidol groups. Incidence of Parkinsonism in the olanzapine LAI study showed no statistically significant difference between drug and placebo (2%–6% olanzapine LAI vs. 6% placebo), nor was there a significant difference between therapeutic oral olanzapine doses and placebo (study 1: 12%-14% oral olanzapine vs. 15% placebo; study 3: 7% oral olanzapine vs. 8% placebo). However, incidence of Parkinsonism in the haloperidol treatment groups was significantly higher than in the therapeutic oral olanzapine groups in both oral study 1 (42% vs. 12%–14%, *p’s* ≤ .001) and study 2 (53% vs. 14%–19%, *p’s* < .001). Akathisia showed a similar pattern, with no significant differences between olanzapine LAI and placebo (1%–6% olanzapine LAI vs. 6% placebo) or therapeutic oral olanzapine and placebo (study 1: 16%-27% oral olanzapine vs. 23% placebo; study 3: 13% oral olanzapine vs. 13% placebo). However, incidence of akathisia in the haloperidol groups was significantly higher than in the therapeutic oral olanzapine groups in both oral study 1 (46% vs. 16%–27%, *p’s* < .05) and study 2 (34% vs. 9%–12%, *p’s* < .01).

### Discontinuation rates

In Table 5, results from all therapeutically dosed olanzapine treatment groups were combined within each of the 4 studies to provide an overall rate of discontinuation compared with the placebo group and the non-therapeutic dose of oral olanzapine (1 mg/day) used in oral studies 2 and 3. The rate of discontinuations from any cause was lower in the olanzapine LAI study (combined dosages of olanzapine LAI) compared with placebo and was also lower than the rates seen in oral olanzapine studies 1 and 2 (combined dosages of oral olanzapine, haloperidol, placebo, and non-therapeutic dose of oral olanzapine) and in oral olanzapine study 3 (oral olanzapine 10 mg/day, non-therapeutic dose of oral olanzapine, and placebo). The rate of discontinuation due to adverse events among patients treated with olanzapine LAI was comparable to patients in the placebo group. Differences in overall discontinuation rate across studies appeared to be driven primarily by higher rates of discontinuation due to lack of efficacy in the oral olanzapine studies 1 and 3 relative to the olanzapine LAI study. The incidence of discontinuation due to an adverse event for oral olanzapine in study 2 (11%) was more than twice that of olanzapine LAI (4%), but no statistical comparisons were made between the studies regarding discontinuation rates.

**Table 5:** Discontinuation rates for the olanzapine long-acting injection and olanzapine oral studies

	**OLZ LAI (N = 404)**		**Oral Study 1 (N = 335)**			**Oral Study 2 (N = 431)**			**Oral Study 3 (N = 152)**		
	**PLC (n = 98)**	**OLZ LAI (n = 306)**^**a**^	**PLC (n = 68)**	**HAL (n = 68)**	**OLZ (n = 198)**^**a**^	**1 mg (n = 88)**	**HAL (n = 81)**	**OLZ (n = 262)**^**a**^	**PLC (n = 50)**	**1 mg (n = 52)**	**OLZ (n = 50)**
**Discontinued (%)**											
All Cause^b^	42.9	31.0	67.6	56.5	56.1	44.3	46.9	37.8	80.0	76.9	62.0
Lack of Efficacy	24.5	11.4	47.1	27.5	32.3	18.2	19.8	14.1	74.0	61.5	56.0
Patient Decision	9.2	11.8	2.9	10.1	10.6	9.1	7.4	7.3	5.8	2.0	2.0
Adverse Event	5.1	4.2	10.3	8.7	5.1	11.4	14.8	10.7	0.0	9.6	4.0
Lost at Follow- up	1.0	0.7	1.5	7.2	3.0	1.1	2.5	1.5	4.0	0.0	0.0

^a^ Therapeutic dose groups were pooled

^b^ All-cause percentages also include discontinuations for reasons other than those shown

Abbreviations: *1 mg*: 1 mg/day non-therapeutic dose of olanzapine; *HAL*: haloperidol; *OLZ*: olanzapine; *LAI*: long-acting injection; *PLC*: placebo

## Discussion

This cross-study, post-hoc analysis indicates that patients with acutely exacerbated schizophrenia treated with olanzapine LAI dosages of 405 mg/4 weeks, 210 mg/2 weeks, and 300 mg/2 weeks had a similar magnitude of symptom reduction as patients treated with 10 ± 2.5 mg/day and 15 ± 2.5 mg/day of oral olanzapine and 15 ± 5 mg/day of oral haloperidol during 6 weeks of acute treatment. Using historical clinical data for oral olanzapine provided a helpful framework for understanding the relative efficacy and tolerability of the long-acting formulation of olanzapine.

### Effect sizes

Importantly, the 4 studies presented in this analysis were not conducted concurrently, and cross-study comparisons should be interpreted with caution. Therefore, effect size calculations were performed to allow a standardized comparison among the 4 studies. In general, an effect size above 0.5 standard deviations is considered “large” [19]. Comparison of effect sizes among the 4 studies suggests that the magnitude of symptom reduction seen with the 3 doses of olanzapine LAI was “large” and generally similar to that of approximately 10 to 15 mg/day oral olanzapine or 15 mg/day oral haloperidol. The evaluation of assessment scores and relative effect sizes in this analysis demonstrate that despite clinical and research changes over the years, the response to treatment with olanzapine LAI as measured against placebo was comparable to results from earlier oral olanzapine clinical trials against placebo.

### Onset of efficacy

The post-hoc exploration of early onset of action based on mean change in symptoms measured by the BPRS or PANSS total scales in each study suggests that speed of onset with olanzapine LAI may be at least as rapid as that of therapeutic doses of oral olanzapine and oral haloperidol. Patients in all 3 treatment groups in the olanzapine LAI study achieved statistically significant reduction in symptoms within 1 week of receiving their first injection, which was comparable to or better than results observed for oral olanzapine in studies 1, 2, and 3. Of note in the olanzapine LAI study, the clinical trial protocol did not allow supplementation with any oral antipsychotic medication, including oral olanzapine. The ability to begin to realize improvement of acute symptoms following the first injection, without the need for additional oral antipsychotic medication for symptom control during the initial weeks or months of treatment, may simplify the treatment plans for patients with acute symptoms.

### Olanzapine plasma concentrations

Pharmacokinetic evaluation indicated that olanzapine plasma concentrations were in the therapeutic range as early as day 3 for the LAI study. Moreover, endpoint concentrations were generally similar across the 4 studies. These findings are consistent with the clinical observations. Interestingly, while all LAI patients had endpoint concentrations within the therapeutic range, some oral patients had concentrations below that range, suggesting that some oral patients may have failed to take their dose.

### Safety and tolerability

Weight gain is very commonly reported during treatment with some atypical antipsychotics such as olanzapine. In this analysis weight gain was most similar between olanzapine LAI and oral olanzapine in oral study 1, with patients gaining a mean of 3.5 to 4 kg on the higher doses of olanzapine. Patients treated with olanzapine LAI or therapeutic doses of oral olanzapine gained significantly more weight than did those treated with haloperidol or placebo. A significantly greater percentage of patients treated with olanzapine LAI or therapeutic doses of oral olanzapine gained clinically significant amounts of weight than did those treated with haloperidol or placebo. Although these were all short-term studies, it is important to note that changes in metabolic parameters have also been reported during long-term treatment with olanzapine [21,22]. Therefore, the potential consequences of weight gain should be considered prior to starting olanzapine treatment. Patients receiving olanzapine should have their weight monitored regularly.

With respect to the development of extrapyramidal symptoms, a significantly greater percentage of patients treated with haloperidol experienced treatment-emergent Parkinsonism or akathisia compared with those treated with olanzapine LAI, oral olanzapine, or placebo. The observed differences between the olanzapine LAI study and the oral studies may be due in part to historical context. At the time that the oral studies were conducted, patients were more likely to have been treated with typical antipsychotics previously, which may have influenced rates of extrapyramidal symptoms in the study despite the washout period. Also, rater expectations regarding extrapyramidal symptoms may have been different at that time, and inclusion of a haloperidol arm may have also biased rater expectations regarding extrapyramidal symptoms.

### Post-injection delirium/sedation syndrome (PDSS)

Although no cases of PDSS occurred during the acute olanzapine LAI study, it is important to note that this is a risk for this depot formulation. During other olanzapine LAI clinical trials, adverse events related to delirium and/or excessive sedation (including coma) were identified in a small percentage of patients following injection. These events have been reported following <0.1% of injections of olanzapine LAI in <2% of patients [23]. Because of the risk of PDSS, safety precautions—including a post-injection observation period— must occur at the time of each injection. Clinicians should consider these factors when weighing the overall risks and benefits of olanzapine LAI for each patient.

### Study discontinuation rates

The low discontinuation rates in the olanzapine LAI study are encouraging considering that discontinuation rates are high in clinical studies of treatments for schizophrenia, but it is important to recognize that discontinuation rates in actual clinical use remain to be determined. Patients who have difficulty adhering to medication schedules are usually not participants in controlled clinical studies [24-26]. Additionally, these studies employed both inpatient and outpatient treatment settings which may have influenced the overall study discontinuation rates. Nevertheless, the present findings suggest possible improvement in treatment persistence for patients who receive a long-acting injection.

### Limitations

These findings are based on 4 separate studies, each analyzed separately, with a span of approximately 10 years between the oral olanzapine studies and the olanzapine LAI study. While effect size calculations help assess the magnitude of efficacy changes and comparison across baselines show similarities of symptoms, there are differences that must be considered when evaluating this information. Patient populations in these studies may have been different, particularly in their previous exposure to medications. Treatment paradigms at the time of the oral studies in the early 1990s were based on the use of first generation antipsychotic medications while current treatment includes the frequent use of second generation antipsychotic agents. It should also be noted that although the present analyses did not exclude any studies meeting our selection criteria, all studies were conducted by Lilly and did not include studies conducted by other sponsors.

It should also be noted that the present analyses are limited in their ability to provide comparisons of specific doses between oral and olanzapine LAI because of the differing length of time needed to reach steady state plasma concentrations for each olanzapine formulation. Olanzapine LAI does not reach steady-state concentrations of olanzapine for at least 3 months, while oral olanzapine should reach steady state levels within the first week of exposure. Therefore, the LAI results seen in the 6-week period examined in this work represent pre-steady state olanzapine levels, which are slightly lower than the levels which will ultimately be achieved over a longer period of time. For instance, while the 405 mg/4 week dose may ultimately correspond to a 15 mg/day oral dose, the short-term results presented here indicate that this dose may provide olanzapine concentrations more similar to a 10 mg/day dose in the first 8 weeks of treatment. This is consistent with the pharmacokinetic and clinical findings from a previous study [27]. An additional consideration is that oral olanzapine studies 1 and 2 allowed semi-flexible dosing of the olanzapine target dose by ±2.5 mg/day while the olanzapine LAI study used strictly fixed doses and prohibited any oral antipsychotic supplementation.

An additional limitation is that only 2 of the 3 oral studies were placebo-controlled, and the 1-mg oral olanzapine reference dose did not perform like placebo with respect to safety measures and some small differences in treatment response. Therefore effect sizes vs. the 1-mg arm are not comparable with effect sizes vs. placebo.

## Conclusions

Patients treated acutely with olanzapine LAI showed a similar pattern of improvement to that seen historically in 3 previous studies of oral olanzapine. Onset of action for the LAI formulation appeared as early as the first week after injection, with olanzapine plasma concentrations appearing similar across the LAI and oral studies. With the exception of injection-related adverse events, the efficacy and tolerability profile of olanzapine LAI is similar to oral olanzapine.

## Abbreviations

BPRS = Brief Psychiatric Rating Scale; DSM = Diagnostic and Statistical Manual of Mental Disorders; LAI = long-acting injection; OLZ = Olanzapine; PANSS = Positive and Negative Syndrome Scale total score; PDSS = Post-injection Delirium/Sedation Syndrome.

## Competing interests

Holland C. Detke, Fangyi Zhao, and Michael M. Witte are full-time employees of Eli Lilly and Company.

## Authors’ contributions

HCD and FZ conceived and designed the analyses; FZ was responsible for statistics. HCD, FZ, and MMW contributed to the interpretation of results and drafting of the manuscript, and all reviewed and approved the final version of the manuscript.

## Pre-publication history

The pre-publication history for this paper can be accessed here:

http://www.biomedcentral.com/1471-244X/12/51/prepub

## Supplementary Material

Additional file 1 **Mean changes in PANSS total scores during six weeks of treatment in olanzapine long-acting injection and oral olanzapine studies of acute schizophrenia.** This file contains a figure depicting the visitwise mean changes in PANSS total scores for the 3 studies. Click here for file
