# Supplementary material for: Efficacy of olanzapine long-acting injection in patients with acutely exacerbated schizophrenia: an insight from effect size comparison with historical oral data
Source: BMC Psychiatry. 2012 May 30;12:51. doi: 10.1186/1471-244X-12-51 (PMC3403915; doi:10.1186/1471-244X-12-51)

Additional file 1. Mean changes in PANSS total scores during six weeks of treatment in olanzapine long-acting injection and oral olanzapine studies of acute schizophrenia

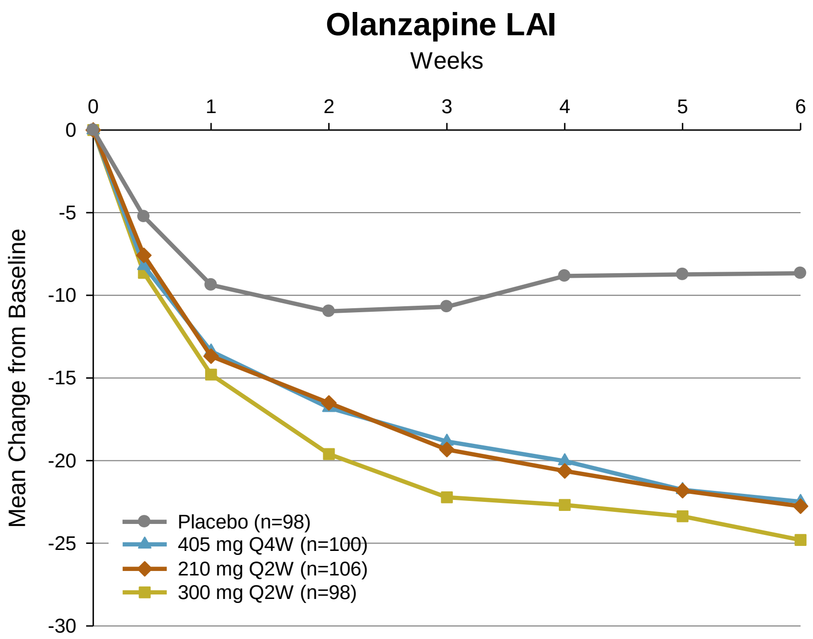

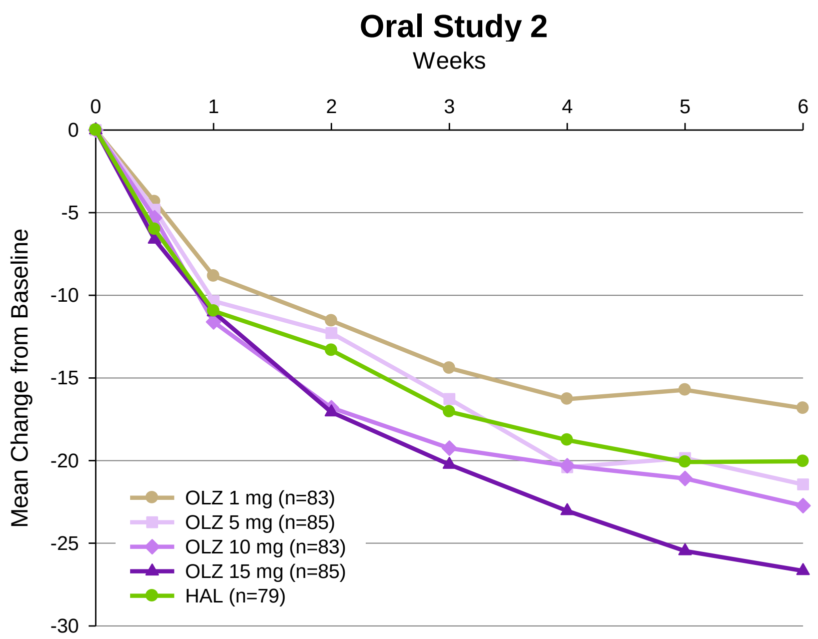

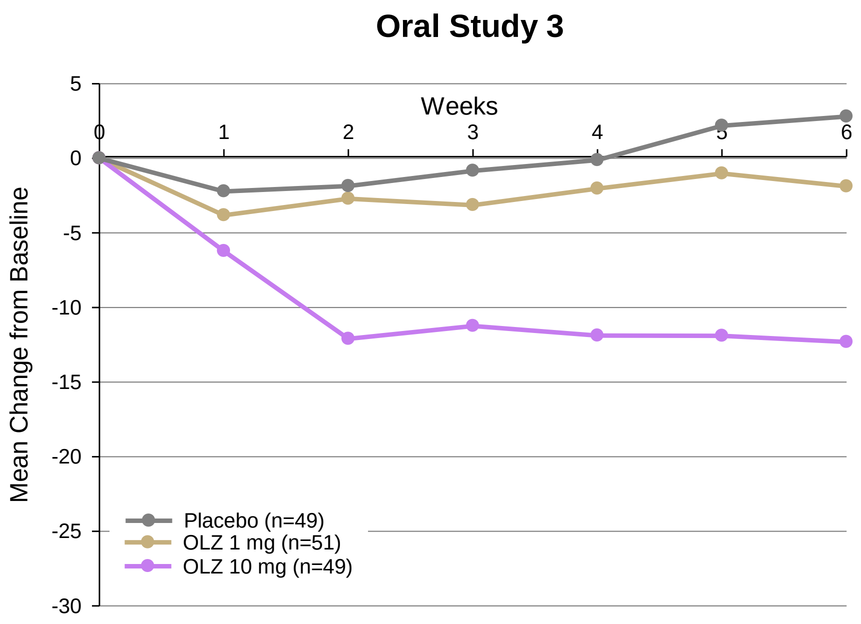

Supplement: Additional file 1 — Mean changes in PANSS total scores during six weeks of treatment in olanzapine long-acting injection and oral olanzapine studies of acute schizophrenia. This file contains a figure depicting the visitwise mean changes in PANSS total scores for the 3 studies. [file 1471-244X-12-51-S1.pdf]
